# Associations between Combined Psychological and Lifestyle Factors with Pain Intensity and/or Disability in Patients with Chronic Low Back Pain: A Cross-Sectional Study

**DOI:** 10.3390/healthcare11222928

**Published:** 2023-11-09

**Authors:** Eirini Tsatsaraki, Izolde Bouloukaki, Georgios Kontakis, Antonis F. Vakis, Maria Basta

**Affiliations:** 1 Nursing Department, University Hospital of Heraklion, University of Crete, 70013 Heraklion, Greece; tsatsaraki.irini@yahoo.gr; 2 Department of Social and Family Medicine, University of Crete, 70013 Heraklion, Greece; izolthi@gmail.com; 3 Department of Orthopaedics and Traumatology, University Hospital of Heraklion, University of Crete, 71110 Heraklion, Greece; 4 Department of Neurosurgery, School of Medicine, University of Crete, 70013 Heraklion, Greece; 5 Division of Psychiatry and Behavioral Science, School of Medicine, University of Crete, 70013 Heraklion, Greece

**Keywords:** chronic low back pain, depression, sleep complaints, anxiety

## Abstract

Chronic low back pain (CLBP) is common in primary care, causing disability and economic burden globally. We aimed to compare socio-demographic, health, lifestyle, and psychological factors in people with and without CLBP and correlate them with clinical outcomes in people with CLBP. A total of 253 volunteers with and 116 without CLBP provided sociodemographic information, daily habits, medical history, subjective sleep complaints (Penn State Sleep Questionnaire), low back pain intensity, and disability (Quebec Back Pain Disability Scale), as well as the Zung Self-Rating Scale for self-assessment of depression and Self-Rating Anxiety Scale. CLBP diagnosis was linked with female gender and older age, as well as a higher level of sleep complaints such as sleepiness, OSA and insomnia symptoms, and a higher prevalence of moderate to severe depressive symptoms. The combination of moderate to severe depressive symptoms with obstructive sleep apnea or insomnia symptoms was the most important predictive factor for functional disability in CBLP patients (OR 13.686, 95% CI 4.581–40.885; *p* < 0.001). In conclusion, depressive symptoms and subjective sleep complaints appear to relate to greater CLBP intensity and/or CLBP-related disability in people with CLBP. A holistic approach is crucial for treating chronic CLBP patients, including psychological and sleep issue assessment and management, to improve their quality of life.

## 1. Introduction

Low back pain (LBP) is the most frequently occurring musculoskeletal disorder globally, with a prevalence of up to 85% throughout one’s lifespan [1]. Being the primary cause of disability worldwide and among the top five reasons for primary care visits [2], LBP leads to significant healthcare costs and lost productivity [3,4]. In Greece, LBP was among the leading five causes of years lived with disability from 2000 to 2016 [5]. Although low back pain is typically self-limited and has a favorable long-term prognosis, some individuals may experience chronic low back pain (CLBP), which is defined as pain that persists for three months or more [6]. CLBP is a prevalent issue in Europe, with 1 in 5 individuals suffering from this condition and experiencing a negative impact on their physical and mental well-being [7,8].

The prevalence of CLBP has raised concerns, with research emphasizing the impact of intricate biological, psychological, and social interplays on CLBP [9,10,11]. The consequences of CLBP surpass mere pain, causing substantial mental health issues, including depression [12,13]. Indeed, individuals with CLBP are more prone to depression than the general population [14,15,16], leading to the utilization of additional healthcare resources and the development of further comorbidities such as diabetes, hypertension, chronic obstructive pulmonary disease, and anxiety [17]. Furthermore, prior investigators have implied that various psychological factors, such as anxiety and depression, are correlated with the intensity of pain and/or disability in individuals with CLBP [18,19,20].

In addition to mood disturbances, disrupted sleep patterns have also been reported in individuals with CLBP [21,22]. A majority of individuals with CLBP experience symptoms of insomnia [23], and poor sleep quality and quantity compared to asymptomatic counterparts [21,24]. On the other hand, individuals suffering from sleep disorders may present higher prevalence of anxiety and depression, suboptimal quality of life [25], and increased severity and duration of chronic pain [25,26]. Notably, earlier studies have shown complex associations among pain, sleep, anxiety, and depression, without a distinct causal order [23,27]. Furthermore, based on a recent review, anxiety, depression, and sleep quality were identified as major contributors to the quality of life of individuals with CLBP [28]. However, the degree to which depression, anxiety, and sleep disorders are associated with CLBP intensity or CLBP-related disability is still not fully understood. A deeper understanding of these associations can enhance the medical care provided to these patients.

To address this gap, we conducted a cross-sectional study aiming to (1) compare comprehensively socio-demographic, health, lifestyle (sleep, physical activity), and psychological factors (depression, anxiety) between people with CLBP and patients with other chronic diseases (without CLBP), and (2) quantify the correlations between these psychological and lifestyle factors, and clinical outcomes (intensity of CLBP and CLBP-related disability) within people with CLBP after considering other confounders. We hypothesized that patients with CLBP compared to other chronically ill people, may present more severe depressive/anxiety and sleep disturbance symptoms and these symptoms combined relate to pain-related and quality-of-life indices.

## 2. Materials and Methods

### 2.1. Study Patients

A cross-sectional study was conducted between February 2019 and May 2021 in consecutive patients visiting the outpatient Department of Orthopedic and Neurosurgery Clinic at the University Hospital of Heraklion, Crete, Greece. To be included, patients had to meet the following criteria: (a) be aged ≥18 years with chronic low back pain, (b) have an education level above elementary school, and (c) provide written informed consent. The control group consisted of patients who sought medical attention in our hospital outpatient departments (internal medicine, surgery, and ophthalmology) that do not assess pain syndromes but had no history of LBP in the last 24 months. Participants were excluded if they refused to participate, had severe neurological or mental disease, were pregnant, had a history of recent lumbar spine fracture or surgery, or had poor understanding of the Greek language.

### 2.2. Data Collection

Participants underwent a thorough evaluation that assessed anthropometric parameters, such as BMI, medical history, and comorbidities, including physician-based diagnosis for depression, smoking history, and alcohol intake. The evaluation lasted around 20–30 min for each participant. All questionnaires used and interviews conducted were in Greek language. Based on the previous literature and semi-structured interviews with a sample of 10 patients, who were conveniently available for participation, we developed a structured questionnaire, which included questions in the following categories: 1. Socio-demographic characteristics (age, gender, family status, education, work status, living arrangements, place of residence); 2. Lifestyle factors (smoking status, alcohol, physical activity); 3. Health status based on self-reports (comorbidities, comorbid musculoskeletal pain conditions); 4. Subjective sleep and sleep problems evaluated using the Penn State Sleep Questionnaire; 5. Functional disability with the Quebec Back Pain Disability Scale; 6. Pain Intensity with Visual Analogue Scale; 7. Depression symptoms using the Zung’s Self-Rating Depression Scale; 8. Questions to assess the impact of pain on mobility, self-care, routine activities, and patient psychology; 9. Stress using the Zung Self-Rating Anxiety Scale.

#### 2.2.1. Penn State Sleep Questionnaire (PSSQ)

Subjective sleep and presence of any sleep disorder were evaluated using 20 sleep-related questions from a standardized questionnaire, used in the Penn State Cohort and described in detail elsewhere [29]. In the current analysis subjective sleep variables examined were non-refreshing sleep, excessive daytime sleepiness (EDS), obstructive sleep apnea (OSA) symptoms and insomnia symptoms qualified in terms of severity on a scale of 0–4 (0 = none, 1 = mild, 2 = moderate, 3 = severe). Non-refreshing sleep was evaluated according to a positive response (“often” or “always”) to the following question: “Do you feel groggy and un-refreshed after you wake up in the morning?”. Excessive daytime sleepiness (EDS) was evaluated according to a positive response (“often” or “always”) to one or both of the questions: “Do you feel groggy or sleepy most of the day but manage to stay awake?”, and/or “Do you have irresistible sleep attacks?”. The presence of symptoms consistent with sleep apnea was indicated by a positive response on one or both of the following questions: “Do you know/Have you been told that you stop breathing or breath irregularly during sleep, occasionally, often or always?” and “Do you know/Have you been told that you snore during sleep to a moderate/severe degree?”. Finally, the presence of “insomnia symptoms” was established with a “yes” answer to the questions: “Do you have difficulty falling asleep?”, “Do you have difficulty staying asleep?” or “Do you wake up in the morning earlier than desired?” often or always.

#### 2.2.2. Quebec Back Pain Disability Scale (QBPDS)

The translated QBPDS focuses on the daily living activities that are commonly impacted by LBP. The twenty items included are classified into six areas: rest/bed, sit/stand, ambulation, handling of large/heavy objects, movement, and bending/stooping. Using these items, patients can rate their activity restrictions on a scale of 0 (not difficult) to 4 (unable to do). By adding responses to the items, a total score between 0 to 100 is obtained, where higher scores indicate more disability [30,31,32].

#### 2.2.3. Zung’s Self-Rating Depression Scale (ZDRS)

The translated ZDRS was used to assess depressive symptoms during the past week [33,34]. It consists of 20 self-rated questions that cover affective, psychological, and somatic symptoms. The individual specifies the frequency with which the symptom is experienced on a 4-point scale ranging from 1 (a little of the time) to 4 (most of the time). The total theoretical range of the score is 20–80. The higher the score on this scale, the more indicative it is of severe depression. The raw scores are transformed into index scores (range 25–100). A SDS score below 50 is considered normal, while scores from 50 to 59 suggest mild depression. Scores between 60–69 suggest moderate to marked depression, and scores of 70 or above suggest severe depression.

#### 2.2.4. The Zung Self-Rating Anxiety Scale (SAS)

The translated SAS is a 20-item self-rating tool that assesses anxiety levels based on 4 symptom groups: cognitive, autonomic, motor, and central nervous system [35,36]. A 4-point Likert-type scale is used to provide answers regarding symptom frequency, with options such as “a little of the time”, “some of the time”, “good part of the time”, and “most of the time”. By adding up the question scores, a final score of 20 to 80 is obtained. High scores indicate high anxiety levels. The raw scores are transformed into index scores (range 25–100) with SAS index score: <45 considered normal, 45–59 indicates mild anxiety, 60–74 indicates moderate anxiety, and ≥75 severe anxiety.

### 2.3. Procedure

The study’s objectives were explained to the selected participants during their visit, and if they agreed to participate, they gave written consent and anonymously filled out the paper-and-pen tool. There was no compensation given for their participation. To minimize the influence of social desirability bias, participants were instructed to place their completed study tools in an opaque box located outside the practice.

The study was conducted according to the guidelines laid down in the Declaration of Helsinki and all procedures involving human subjects/patients were approved by the Bioethics Committee of the University Hospital of Heraklion, Crete (Protocol Number: 13607/21-11-2018). Written informed consent was obtained from all subjects.

### 2.4. Statistical Analysis

A study involving 77 participants was conducted as a pilot to determine the sample size. Based on the data acquired from the pilot study, a sample size of at least 189 individuals was determined to achieve a minimum of 80% power in detecting a significant difference in measured parameters between the controls and CLBP groups, while accounting for a type-I error rate of 0.05.

Data were analyzed using SPSS software (version 25, SPSS Inc., Chicago, IL, USA). For comparisons between groups, a two-tailed *t*-test for independent samples (for normally distributed) or a Mann–Whitney U test (for non-normally distributed data) was utilized for continuous variables and the chi-square test for categorical variables. To assess whether participants’ baseline characteristics and questionnaires scores could predict the presence and intensity of CLBP and CLBP-related disability, analyses were conducted using logistic regression in 3 steps. First, we performed a series of bivariate analyses to assess the odds ratios (ORs) of each baseline variable (bivariate “bivariate model”). Second, we repeated the analyses with all baseline variables simultaneously entered into the binomial model (the “complete model”), after controlling for potential explanatory variables, including age, sex, BMI, level of education, marital status, physical activity, and major chronic conditions. Finally, we built a “parsimonious model” in which we excluded nonsignificant predictors with no incremental predictive power from the complete model in a stepwise procedure. We checked multicollinearity among the predictors using collinearity statistics to ensure that collinearity between predictor variables was in the acceptable range as indicated by the tolerance value variance inflation factor. Age was considered continuously and categorically (age groups of 18–49, 50–64, and ≥65 years). BMI was also considered continuously and categorically, as BMI groups of <30 and ≥30 kg/m^2^. The Cronbach’s alpha coefficient and item–total correlations were employed to assess the internal consistency of the questionnaires. Spearman’s (rho) coefficients were utilized to compute correlations among the sub-scales of the questionnaires. The test–retest reliability of the aforementioned questionnaire scores was assessed using the intraclass correlation coefficient, ICC. Results were considered significant when *p* values were <0.05.

## 3. Results

### 3.1. Demographic Data

A total of 402 patients were approached to participate and 382 accepted (response rate 95%). After excluding for missing data, a total of 369 participants (253 CLBP patients and 113 controls) were finally included in the study. Table 1 presents the sociodemographic characteristics of the total sample, while Tables S1 and S2 present them separately according to sex. The age group ≥ 65 years was the most frequent, making up 35% of the total sample. The majority of the sample subjects were females (56%), and 69% were married or in a relationship. About a quarter of the participants did not finish high school, while 19% claimed to live alone. Most participants displayed behavioral characteristics of being either non-smokers or former smokers (61%), having low alcohol consumption (80%), and being physically inactive (70%).

When we stratified our sample by sex, we found that the prevalence of CLBP among females was significantly higher in all age groups compared to controls; on the other hand, in males CLBP prevalence was significantly higher only in the age group ≥ 65 years compared to controls. The demographic data by diagnostic group, i.e., CLBP patients and controls, are presented in Table 1. Both groups showed no significant differences in BMI, marital status, smoking, education level, presence of coronary artery disease, and congestive heart failure; however, there were variations in sex, age, alcohol use, presence of other comorbidities, physical activity, and occupational status between the groups.

### 3.2. Psychological and Sleep Parameters

Psychological and sleep parameters are presented in Table 2 for the total group and by diagnostic groups, while Tables S3 and S4 present the data separately for each sex. The Cronbach’s alpha coefficients for Quebec, SDS, and SAS scores in this specific population were calculated as 0.678, 0.491, and 0.581, respectively. The questionnaires demonstrated ICC values of 0.528, 0.342, and 0.517, respectively. The global scores of all questionnaires were significantly correlated with each component (*p* < 0.01). Item–total Spearman’s rho correlations ranged from 0.711 (component 4) to 0.838 (component 1) for Quebec, from 0.425 (component 16) to 0.525 (component 10) for SDS and from 0.520 (component 18) to 0.623 (component 1) for SAS.

People with CLBP demonstrated significantly higher pain intensity and disability than controls (*p* < 0.05). The effect of pain on all factors examined, i.e., mobility, self-reliant, usual activity, pain/discomfort, and anxiety/depression were significantly higher in the CLBP group compared to controls. After examining the data based on sex, comparable results were obtained. Within the CLBP group, 99% percent reported clinically significant pain/discomfort, whereas 83% and 90% had clinically significant impact on mobility and anxiety/depression, respectively. Both diagnostic groups also exhibited varying symptoms of depression and anxiety. It is worth noting that nearly all participants scored outside the normal range for anxiety (SAS). Additionally, in the total group, 42% of participants reported moderate to severe depressive symptoms, while only 21% had a physician diagnosis of depression and were taking medications. The prevalence of insomnia symptoms, OSA symptoms, and sleepiness were 52%, 46%, and 38%, respectively. CLBP patients reported significantly higher prevalence of insomnia (64% vs. 26%, *p* < 0.001) and OSA symptoms (51% vs. 35%, *p* = 0.006). As expected, females displayed a higher prevalence of insomnia symptoms in contrast to males who displayed a higher prevalence of OSA symptoms (Tables S3 and S4). Also, comorbid moderate to severe depressive symptoms were significantly greater in CLBP patients than in controls an association that persisted only in the female population. Additionally, in both males and females, CLBP patients had significantly greater OSA and insomnia symptoms than controls.

### 3.3. Factors Correlating with the Presence of CLBP

Table 3 contains the condensed results of various independent multivariate analyses. Each analysis controlled for age, gender, BMI, level of education, marital status, and major chronic conditions while examining the independent impact of CLBP on various outcome variables. This table reveals that having CLBP was associated with female sex and older age, as well as worse physical and mental health outcomes measured by (i) a higher level of sleep symptoms such as sleepiness, OSA and insomnia symptoms, and (ii) a higher prevalence of physician-diagnosed depression, and depressive symptoms. Additional analysis also revealed a significant association of combined moderate to severe depressive and sleep disorders symptoms (OSA or insomnia symptoms) with CLBP presence (OR 6.291). Similar results were obtained from conducting separate analyses for both males and females.

### 3.4. Correlations between Pain Intensity and Disability with Demographic, Psychological, and Sleep Parameters in CLBP Patients

The level of functional disability for CBLP patients (Quebec score ≥ 33) was independently associated with age ≥ 65, physician diagnosed depression, educational status, SDS score, moderate to severe depressive symptoms (SDS) score ≥ 60 and OSA symptoms (Table 4). The relationship between functional disability and depressive symptoms was more prominent in females for CBLP patients compared to the association with OSA symptoms, which is only evident in males. The combination of moderate to severe depressive symptoms with sleep disorders symptoms was the strongest predictive factor for functional disability for CBLP patients, and this finding was more prominent in the female population.

## 4. Discussion

The study looked into associations between CLBP presence, intensity, and related disability, and a range of socio-demographic, health, lifestyle (sleep, physical activity), and psychological factors (depression, anxiety). The main findings of the study were that CLBP patients are more likely to be females, over 65 years of age, with low educational status and physical activity, and reporting moderate to severe depressive symptoms and subjective sleep disturbances compared to other chronically ill patients. Furthermore, the study indicates that among CLBP patients, pain-related and quality-of-life indices are related to older age, physician diagnosed depression, lower educational status, and moderate to severe depressive and subjective sleep disturbances.

Several health conditions may influence the presence of CLBP, and common mental disorders, such as depression, seem to be associated with an increased prevalence of CLBP [37]. Indeed, the relationship between CLBP and depression has been well-documented in previous studies [38,39,40]. Notably, patients with chronic pain in orthopedic or rheumatology clinics may have major depression prevalence ranging from 21 to 89% [41]. Additionally, a systematic review that assessed 25 cohort studies found that depression was the most commonly observed prognostic risk factor for CLBP [42]. Likewise, in another systematic review of 10 observational studies, a moderate association was found between depression and anxiety and high levels of pain and disability in CLBP patients [43]. Converging evidence also points to the fact that depressive symptoms can make pain intensity, disability, and treatment outcomes worse in patients with chronic LBP, leading to a vicious cycle of LBP and depression [14,44]. Unlike our hypothesis, anxiety symptoms before adjustments for confounders in the CLBP population were less prevalent compared to other chronically ill patients of our control group. Given that the control group included patients with various chronic conditions, it would be prudent to speculate that anxiety might be strongly correlated with health problems other than CLBP.

However, little is known about the extent of comorbid CLBP and depression in the Greek population. An earlier Greek cross-sectional study conducted in an urban setting found that depressed participants reported 2.3-times-higher LBP severity than those without depression [44]. In a more recent small study, no significant association was noted between depressive symptoms and pain, disability, and the health-related quality of life of CLPB patients [45]. Our study shows that 28% of the CLBP population suffer from clinically diagnosed comorbid depression, and 43% report moderate to severe depressive symptoms. Patients with moderate to severe depressive symptoms reported 2.5-times-higher CLBP prevalence and 11.8-times-higher functional disability than those without. It is plausible that when someone has CLBP, their inability to work and engage in social activities potentially leads to a sense of helplessness and despair, which often results in depression [46]. On the other hand, we found no correlation between anxiety symptoms and the presence or functional disability of CLBP. This can be attributed to the fact that practically all individuals (99%) with or without CLBP in our sample experienced some level of anxiety symptoms.

CLBP can be also influenced by lifestyle factors such as sleep problems and amount of physical activity, which may exacerbate depression and pain. This was also the case in our study, which also found a correlation between CLBP and lower physical activity, subjectively assessed OSA, and insomnia symptoms. In line with our results, the evidence suggests that over half of individuals with LBP experience sleep disturbances [47,48]. Patients with OSA symptoms in our study also reported 6.3-times-higher CLBP functional disability than those without. Although the mechanism underlying the association between sleep disturbance and pain is still unclear, the attribution of this finding may be linked to the decreased pain threshold resulting from sleep disturbance, as evidenced by experimental studies [49]. In addition, earlier studies have pointed out that sleep disorders and decreased physical activity could aggravate the symptoms of CLBP [50,51,52].

A novel finding of our study was the relationship between the combined effect of depressive with OSA or insomnia symptoms and the occurrence of CLBP. Moreover, if CLBP patients experience moderate to severe depressive symptoms in addition to sleep disturbances such as OSA or insomnia symptoms, their probability of functional disability increases by approximately 13 times, with this observation being more significant among females. The current study’s observation may be accounted for by the possibility that negative emotions can intensify the impact of sleep disturbances on pain, potentially by increasing arousal or disrupting circadian rhythms [53]. However, the relationship between depression, sleep disorders, and CLBP has been minimally explored in previous research. A recent study highlighted that females with common mental disorders and insufficient sleep quality were more predisposed to CLBP [54].

The implications of this study are significant, as CLBP has a substantial financial impact on public health and health systems. Improved awareness of the interplay between depression, sleep disturbances, and CLBP can guide management of CLBP among patients and healthcare providers and enhance the clinical care of these patients. Accordingly, among patients with CLBP it is crucial to conduct frequent routine screening, especially in primary care settings, for signs of depression and sleep disruptions, as these symptoms may develop over time. By conducting an early assessment in a comprehensive way of depressive and sleep symptoms, general practitioners can identify patients who are at risk of disability and poor recovery and provide patient-centered care. Early referral to multi-disciplinary care may be appropriate for this population. Therapeutic protocols that take into account sex, the psychological profile, and sleep history of the patient might prove to be more efficacious than those that solely concentrate on physical symptoms like pain and disability in the treatment of CLBP. Furthermore, interventions should encompass a holistic approach. Apart from analgesics, or surgical intervention, psychotherapeutic intervention may also be considered and applied accordingly. Such interventions may include cognitive behavioral therapy for depression and anxiety as well as insomnia symptoms. Additionally, a thorough diagnosis and management of OSA, may be essential. The information revealed in the current study might also assist in constructing healthcare approaches for individuals with CLBP and function as a standard for future specific public health campaigns.

To the best of our knowledge, the study represents one of the first studies of its kind conducted in Greece, deriving from a south European population with special sociodemographic and cultural characteristics. Also, our study explored in a comprehensive way the interrelationship between CLBP, depression, and sleep disturbances. A relatively large sample size of adult men and women, the use of previously tested and validated instruments to evaluate the presence of subjective sleep problems, depressive, and anxiety symptoms, and also including a control group are among the study’s strengths. Additionally, it is important to note that multivariate analyses were conducted to examine the links between depressive, OSA and insomnia symptoms, and CLBP, while accounting for various confounding variables. Notwithstanding, a few limitations of this research ought to be mentioned. Firstly, the study design poses a limitation given that cross-sectional designs are constrained in establishing causal connections between CLPB, depressive symptoms, and sleep disturbances. Secondly, due to the self-reported nature of the data, including affective symptoms and sleep disorders, there may be a potential for information bias, such as social desirability and recall bias. Thirdly, approaching patients at random for participation may have inadvertently caused selection bias. Moreover, the study’s sample, having been taken solely from a hospital in Heraklion, may not be representative of the general population in Greece. As a result, the generalization of the results to CLBP patients in other clinical settings or Greek regions should be approached with caution. Furthermore, a part of this study was carried out at the initial phase of the COVID-19 pandemic. This could potentially impact the sample size due to the reduction in in-person appointments. Nevertheless, the majority of our data were gathered prior to the onset of the COVID-19 pandemic, with only a limited amount from that timeframe. Finally, our control group was not matched in terms of sex, age, alcohol use, presence of other comorbidities, physical activity, and occupational status with the CLBP group so statistical corrections were made. Also, the control group consisted of chronically ill participants attending the outpatient clinics of the hospital and comorbid pain syndromes, other than CLBP, cannot be excluded. Future prospective cohort studies in larger populations also including healthy matched controls are necessary to gain a deeper understanding of those correlations at a national level.

## 5. Conclusions

In conclusion, our results indicate a significant relationship between depressive symptoms, sleep disturbances, and CLBP. The combination of depressive symptoms with symptoms of OSA or insomnia emerged as the most noteworthy prognostic factor for functional disability in CLPB patients. Therefore, addressing sleep disruptions in the correlation between depression and CLBP may enable the successful implementation of preventive actions, especially in primary care settings, that lower the frequency of new pain episodes and elevate the quality of life of individuals suffering from CLBP. However, we underscore the importance of further longitudinal investigations to probe the causal link between depression, sleep disturbances, and CLBP.

## Figures and Tables

**Table 1:** Demographic and medical characteristics of participants with chronic low back pain (CLBP) and controls.

Characteristic	Total (*n* = 369)	Controls (*n* = 116)	CLBP (*n* = 253)	*p*-Value
**Sex**				
Μales	164 (44%)	68 (59%)	96 (38%)	<0.001
**Age**				
Age, years	57 ± 15	51 ± 13	59 ± 15	<0.001
Age group 18–49 years	121 (33%)	46 (40%)	75 (30%)	
Age group 50–64 years	120 (32%)	54 (46%)	66 (26%)	
Age group ≥ 65 years	128 (35%)	16 (14%)	112 (44%)	<0.001
**ΒΜΙ**	28 ± 4	28 ± 4	28 ± 4	0.553
>30 kg/m^2^	88 (24%)	30 (27%)	58 (23%)	0.374
**Smoking status**				
Current	143 (39%)	45 (39%)	98 (39%)	
Never/Former smoker	226 (61%)	71 (61%)	155 (61%)	0.992
**Frequent Alcohol use**				
(≥1 drink/day)	74 (20%)	13 (11%)	61 (24%)	0.004
**Physical activity**				
Yes (at least 1/week)	111 (30%)	47 (41%)	64 (25%)	
No	258 (70%)	69 (59%)	189 (75%)	0.003
**Level of education**				
Primary level or less	91 (25%)	27 (23%)	64 (25%)	
Secondary level	126 (34%)	32 (28%)	94 (37%)	
Higher level	152 (41%)	95 (49%)	95 (38%)	0.088
**Occupational status**				
Unemployed	86 (27%)	17 (16%)	69 (34%)	
Employed	186 (59%)	87 (80%)	99 (48%)	
Retired	43 (14%)	5 (4%)	38 (18%)	<0.001
**Manual work**	120 (35%)	47 (43%)	73 (32%)	0.047
**Marital Status**				
Married/Partner	254 (69%)	82 (71%)	172 (68%)	0.602
Unmarried/divorced/widowed	115 (31%)	34 (29%)	81 (32%)	
**Living arrangements**				
Living alone	70 (19%)	22 (19%)	48 (19%)	
Living with others	299 (81%)	94 (81%)	205 (81%)	0.999
**Co-morbidities**				
Arterial hypertension	141 (39%)	28 (24%)	113 (47%)	<0.001
COPD	20 (6%)	2 (2%)	18 (7%)	0.029
Diabetes type 2	73 (20%)	13 (11%)	60 (25%)	0.003
Chronic cerebrovascular disease	10 (3%)	0 (0%)	10 (5%)	0.018
Coronary artery disease	36 10%)	14 (12%)	22 (9%)	0.359
Congestive heart failure	15 (4%)	5 (4%)	10 (4%)	0.924
Depression (on medication)	73 (21%)	7 (6%)	66 (28%)	<0.001
**Comorbid musculoskeletal pain conditions**	84 (28%)	16 (14%)	68 (36%)	<0.001

**Table 2:** Summary of scores of pain, psychological and subjective sleep variables by diagnostic group.

Characteristic		Total (*n* = 369)	Controls (*n* = 116)	CLBP (*n* = 253)	*p*-Value
**Variables**	**Measures**				
**Pain Intensity**	**VAS**	4.2 ± 2.2	3.0 ± 1.7	4.8 ± 2.2	<0.001
	**VAS ≥ 6**	113 (32%)	17 (15%)	96 (39%)	<0.001
**Low back pain-related disability**	**QBPDS**	29 (16, 48)	7 (3, 16)	42 (27, 58)	<0.001
	**QBPDS ≥ 50**	87 (25%)	0 (0%)	87 (36%)	<0.001
**Effect of pain on**					
Mobility	Minor/major problem	233 (65%)	29 (25%)	204 (83%)	<0.001
Self-reliance	Minor/major problem	98 (27%)	8 (7%)	90 (37%)	<0.001
Usual activity	Minor/major problem	206 (57%)	33 (29%)	173 (70%)	<0.001
Pain/Discomfort	Minor/major problem	327 (91%)	84 (73%)	243 (99%)	<0.001
Anxiety/depression	Minor/major problem	306 (85%)	84 (73%)	222 (90%)	<0.001
**Depression**	**SDS**	58 ± 9	56 ± 11	59 ± 8	0.005
Normal range	25–49	20 (6%)	9 (8%)	11 (5%)	
Mildly depressed	50–59	189 (53%)	59 (53%)	130 (53%)	
Moderately depressed	60–69	104 (29%)	36 (32%)	68 (28%)	
Severely depressed	≥70	46 (13%)	9 (8%)	37 (15%)	0.158
**Anxiety**	**SAS**	63 ± 9	65 ± 7	62 ± 9	0.006
Normal Range	<45	1 (1%)	0 (0%)	1 (1%)	
Mild to moderate anxiety levels	45–59	108 (31%)	18 (16%)	90 (38%)	
Marked to severe anxiety levels	60–74	213 (61%)	82 (75%)	131 (55%)	
Extreme anxiety levels	≥75	28 (8%)	10 (9%)	18 (8%)	<0.001
**Sleep**	**Insomnia** **symptoms**	191 (52%)	30 (26%)	161 (64%)	<0.001
	**OSA symptoms**	167 (46%)	40 (35%)	127 (51%)	0.006
	**Daytime Sleepiness**	138 (38%)	17 (15%)	121 (48%)	<0.001
**SDS ≥ 60 combined with OSA or insomnia symptoms**		97 (27%)	16 (14%)	81 (33%)	<0.001

VAS: Visual Analogue Scale; QBPDS: Quebec Back Pain Disability Scale; SDS: Zung Self-Rating Depression Scale; SAS: Zung Self-Rating Anxiety Scale.

**Table 3:** Multiple stepwise logistic regression analysis of the relationship between CLBP and various independent variables.

Variable	B	S.E.	*p*-Value	OR (95%CI)
**Females vs. Males**	1.149	0.266	<0.001	3.154 (1.873–5.312)
**Age group** 18–49 years				Ref
**Age group** 50–64 years	0.073	0.310	0.815	1.075 (0.586–1.975)
**Age group** ≥ 65 years	2.302	0.411	<0.001	9.990 (4.463–22.362)
**BMI ≥ 30 kg/m^2^**	−0.546	0.315	0.083	0.579 (0.312–1.075)
**Level of education**				
Primary level or less				Ref
Secondary level	1.257	0.412	0.002	3.516 (1.569–7.876)
Higher level	0.777	0.391	0.047	2.174 (1.010–4.678)
**Living with others**	0.689	0.288	0.589	1.168 (0.664–2.054)
**Married vs. single**	0.155	0.359	0.572	1.225 (0.606–2.478)
**Physical activity**				
(at least 1/week)	−0.816	0.286	0.004	0.442 (0.252–0.775)
**Frequent alcohol use**	0.588	0.364	0.106	1.800 (0.882–3.672)
**COPD**	1.153	0.799	0.149	3.167 (0.661–15.171)
**Hypertension**	−0.242	0.325	0.456	0.785 (0.416–1.484)
**Type 2 diabetes**	0.701	0.400	0.080	2.015 (0.919–4.416)
**Depression**	1.314	0.452	0.004	3.722 (1.534–9.032)
**Sleepiness**	1.175	0.347	0.001	3.239 (1.641–6.392)
**OSA symptoms**	0.729	0.283	0.010	2.073 (1.191–3.607)
**Insomnia symptoms**	1.309	0.288	<0.001	3.704 (2.105–6.518)
**Depressive symptoms** (SDS score)	0.080	0.020	<0.001	1.083 (1.042–1.125)
**SDS ≥ 60**	0.935	0.303	0.002	2.548 (1.409–4.611)
**Comorbid moderate to severe depressive symptom (SDS ≥ 60) and OSA or insomnia symptoms**	1.839	0.397	<0.001	6.291 (2.892–13.685)
**Anxiety (SAS score)**	−0.026	0.017	0.135	0.974 (0.941–1.008)
**SAS score ≥ 60**	−0.886	0.514	0.085	0.412 (0.151–1.130)

**Table 4:** Multiple stepwise logistic regression analysis of the relationship between functional disability for CBLP patients (Quebec score ≥ 33) and demographic, psychological, and sleep parameters in CLBP patients.

	Total (*n* = 253)		Male Population (*n* = 96)		Female Population (*n* = 157)	
Variable	*p*-Value	OR (95%CI)	*p*-Value	OR (95%CI)	*p*-Value	OR (95%CI)
**Age group** ≥ 65 years	0.011	4.425 (1.414–13.848)	0.153	0.098 (0.004–2.379)	0.017	2.851 (0.743–10.944)
Educational level	0.041	0.182 (0.036–0.931)	0.650	0.635 (0.089–4.514)	0.440	2.134 (0.312–14.619)
**Depression**	0.001	7.503 (2.215–25.415)	0.595	1.728 (0.231–12.941)	0.034	3.656 (1.105–12.098)
**OSA symptoms**	<0.001	3.643 (1.855–7.154)	<0.001	17.432 (3.762–80.772)	0.601	1.270 (0.519–3.105)
**Depressive symptoms** (SDS score)	<0.001	1.140 (1.068–1.217)	0.698	1.021 (0.921–1.131)	0.001	1.178 (1.067–1.300)
**SDS ≥ 60**	<0.001	11.749 (4.401–31.366)	0.043	5.874 (0.976–35.345)	<0.001	10.732 (3.039–37.393)
**Comorbid moderate to severe depressive symptom (SDS ≥ 60) and OSA or insomnia symptoms**	<0.001	13.686 (4.581–40.885)	0.038	5.456 (0.944–31.548)	<0.001	11.411 (2.921–44.575)

## Data Availability

The datasets generated during and/or analyzed during the current study are available from the corresponding author on reasonable request.

## Supplementary Materials

The following supporting information can be downloaded at: https://www.mdpi.com/article/10.3390/healthcare11222928/s1.
