# Supplementary material for: Associations between Combined Psychological and Lifestyle Factors with Pain Intensity and/or Disability in Patients with Chronic Low Back Pain: A Cross-Sectional Study
Source: Healthcare (Basel). 2023 Nov 9;11(22):2928. doi: 10.3390/healthcare11222928 (PMC10671559; doi:10.3390/healthcare11222928)
Supplement: Supplementary file 1 [file healthcare-11-02928-s001.zip › healthcare-2661653-supplementary.pdf]

Table S1. Demographic and medical characteristics of participants with chronic low back pain (CLBP) and controls in the male population.

Characteristic	Total (n=164)	Controls (n=68)	CLBP (n=96)	P-Value
**Age**				
Age, years	58 ± 15	49 ± 13	65± 12	<0.001
Age group 18-49 years	37 (22%)	27 (40%)	10 (10%)	
Age group 50–64 years	57 (35%)	34 (50%)	23 (24%)	
Age group ≥ 65 years	70 (43%)	7 (10%)	63 (66%)	<0.001
**BMI**	28 ± 3	28 ± 4	28 ± 3	0.144
>30 kg/m2	32 (20%)	14 (22%)	18 (20%)	0.664
**Smoking status**				
Current	68 (42%)	26 (38%)	42 (44%)	
Never/Former smoker	96 (58%)	42 (62%)	54 (56%)	0.480
**Frequent Alcohol use**				
(≥1 drink/day)	42 (26%)	6 (9%)	36 (38%)	<0.001
**Physical activity**				
Yes (at least 1/week)	47 (29%)	23 (34%)	24 (25%)	
No	117 (71%)	45 (66%)	72 (75%)	0.218
**Level of education**				
Primary level or less	43 (26%)	17 (25%)	26 (27%)	
Secondary level	42 (26%)	12 (18%)	30 (31%)	
Higher level	79 (48%)	39 (57%)	40 (42 %)	0.083
**Occupational status**				
Unemployed	34 (21%)	2 (3%)	32 (35%)	
Employed	101 (64%)	64 (95%)	37 (40%)	
Retired	24 (15%)	1 (2%)	23 (25%)	<0.001
**Manual work**	64 (40%)	30 (46%)	34 (36%)	0.218
**Marital Status**				
Married/Partner	122 (74%)	47 (69%)	75 (78%)	
Unmarried/divorced/widowed	42 (26%)	21 (31%)	21 (22%)	0.193
**Living arrangements**				
Living alone	41 (25%)	17 (25%)	24 (25%)	
Living with others	123 (75%)	51 (75%)	72 (75%)	0.999
**Co-morbidities**				
Arterial Hypertension	76 (47%)	15 (22%)	61 (65%)	<0.001
COPD	13 (8%)	2 (3%)	11 (12%)	0.045
Diabetes Type 2	35 (22%)	7 (10%)	28 (30%)	0.003
Chronic cerebrovascular disease	1 (1%)	0 (0%)	1 (1%)	0.367
Coronary artery disease	19 (12%)	11 (16%)	8 (9%)	0.125
Congestive heart failure	7 (4%)	3 (5%)	4 (4%)	0.957
Depression (on medication)	23 (14%)	3 (5%)	20 (21%)	0.003
**Comorbid musculoskeletal pain conditions**	33 (24%)	11 (17%)	22 (29%)	0.084

Table S2. Demographic and medical characteristics of participants with chronic low back pain (CLBP) and controls in the female population.

Characteristic	Total (n=205)	Controls (n=48	CLBP (n=157)	P-Value
**Age**				
Age, years	55 ± 15	54 ± 12	56± 16	0.332
Age group 18-49 years	84 (41%)	19 (39%)	65 (41%)	
Age group 50–64 years	63 (31%)	20 (42%)	43 (28%)	
Age group ≥ 65 years	58 (28%)	9 (19%)	49 (31%)	0.106
**BMI**	28 ± 5	28 ± 5	28 ± 5	0.860
>30 kg/m2	56 (28%)	16 (36%)	40 (26%)	0.183
**Smoking status**				
Current	75 (37%)	19 (40%)	56 (36%)	
Never/Former smoker	130 (63%)	29 (60%)	101 (64%)	0.622
**Frequent Alcohol use**				
(≥1 drink/day)	32 (16%)	7 (15%)	25 (16%)	0.823
**Physical activity**				
Yes (at least 1/week)	64 (31%)	24 (50%)	40 (26%)	
No	141 (69%)	24 (50%)	117 (74%)	0.001
**Level of education**				
Primary level or less	48 (23%)	10 (21%)	38 (24%)	
Secondary level	84 (41%)	20 (42%)	64 (41%)	
Higher level	73 (36%)	18 (38%)	55 (35%)	0.883
**Occupational status**				
Unemployed	52 (33%)	15 (35%)	37 (33%)	
Employed	85 (55%)	23 (55%)	62 (54%)	
Retired	19 (12%)	4 (10%)	15 (13%)	0.806
**Manual work**	56 (31%)	17 (39%)	39 (29%)	0.226
**Marital Status**				
Married/Partner	132 (64%)	35 (73%)	97 (62%)	0.159
Unmarried/divorced/widowed	73 (36%)	13 (27%)	60 (38%)	
**Living arrangements**				
Living alone	29 (14%)	5 (10%)	24 (15%)	
Living with others	176 (86%)	43 (90%)	133 (85%)	0.397
**Co-morbidities**				
Arterial Hypertension	65 (33%)	13 (27%)	52 (35%)	<0.001
COPD	7 (4%)	0 (0%)	7 (5%)	0.126
Diabetes Type 2	38 (19%)	6 (13%)	32 (22%)	0.170
Chronic cerebrovascular disease	9 (5%)	0 (0%)	9 (7%)	0.062
Coronary artery disease	17 (9%)	3 (6%)	14 (9%)	0.500
Congestive heart failure	8 (4%)	2 (4%)	6 (4%)	0.966
Depression (on medication)	50 (27%)	4 (8%)	46 (33%)	<0.001
**Comorbid musculoskeletal pain conditions**	51 (32%)	5 (11%)	46 (40%)	<0.001

Table S3. Summary of scores of pain, psychological and subjective sleep variables by diagnostic group in the male population.

Characteristic		Total (n=164)	Controls (n=68)	CLBP (n=96)	P-Value
**Variables**	**Measures**				
**Pain Intensity**	**VAS**	3.7 ± 2.0	2.6 ± 1.7	4.4 ± 1.9	<0.001
	**VAS≥6**	38 (24%)	8 (12%)	30 (33%)	<0.001
**Low back pain-related disability**	**QBPDS**	28 (4, 48)	4 (0, 14)	41 (29, 58)	<0.001
	**QBPDS≥50**	31 (19%)	0 (0%)	31 (33%)	<0.001
**Effect of pain on**					
Mobility	Minor /major problem	89 (56%)	16 (24%)	73 (79%)	<0.001
Self-reliant	Minor /major problem	43 (27%)	7 (10%)	36 (38%)	<0.001
Usual activity	Minor /major problem	75 (47%)	12 (18%)	63 (67%)	<0.001
Pain/Discomfort	Minor /major problem	138 (86%)	46 (69%)	92 (98%)	<0.001
Anxiety/depression	Minor /major problem	128 (80%)	45 (67%)	83 (88%)	0.001
**Depression**	**SDS**	59 ± 8	59 ± 7	59 ± 9	0.834
Normal Range	25-49	8 (5%)	5 (7%)	3 (3%)	
Mildly Depressed	50-59	85 (53%)	31 (46%)	54 (57%)	
Moderately Depressed	60-69	45 (28%)	25 (37%)	20 (21%)	
Severely Depressed	≥70	24 (15%)	7 (10%)	17 (18%)	0.058
**Anxiety**	**SAS**	64 ± 10	66 ± 8	63 ± 11	0.119
Normal Range	<45	0 (0%)	0 (0%)	0 (0%)	
Mild to moderate anxiety levels	45-59	49 (31%)	9 (15%)	40 (43%)	
Marked to severe anxiety levels	60-74	84 (54%)	44 (71%)	40 (43%)	
Extreme anxiety levels	≥75	23 (15%)	9 (15%)	14 (15%)	<0.001
**Sleep**	**Insomnia symptoms**	69 (43%)	20 (29%)	49 (52%)	0.004
	**OSA symptoms**	88 (54%)	29 (43%)	59 (63%)	0.011
	**Daytime Sleepiness**	67 (41%)	12 (18%)	55 (59%)	<0.001
**SDS≥60 combined with OSA or insomnia symptoms**		40 (25%)	12 (18%)	28 (30%)	0.037

VAS: Visual Analogue Scale, QBPDS: Quebec Back Pain Disability Scale, SDS: Zung Self-Rating Depression Scale, SAS: Zung Self-Rating Anxiety Scale.

Table S4. Summary of scores of pain, psychological and subjective sleep variables by diagnostic group in the female population.

Characteristic		Total (n=205)	Controls (n=48	CLBP (n=157)	P-Value
**Variables**	**Measures**				
**Pain Intensity**	**VAS**	4.7 ± 2.3	3.6 ± 1.7	4.9 ± 2.3	<0.001
	**VAS≥6**	75 (38%)	9 (20%)	66 (43%)	0.003
**Low back pain-related disability**	**QBPDS**	30 (18, 52)	13 (6, 18)	43 (23, 60)	<0.001
	**QBPDS≥50**	56 (29%)	0 (0%)	56 (38%)	<0.001
**Effect of pain on**					
Mobility	Minor /major problem	144 (72%)	13 (27%)	131 (86%)	<0.001
Self-reliant	Minor /major problem	55 (28%)	1 (2%)	54 (36%)	<0.001
Usual activity	Minor /major problem	131 (66%)	21 (44%)	110 (72%)	<0.001
Pain/Discomfort	Minor /major problem	189 (95%)	38 (79%)	151 (99%)	<0.001
Anxiety/depression	Minor /major problem	178 (89%)	39 (81%)	139 (91%)	0.049
**Depression**	**SDS**	58 ± 10	53 ± 14	60 ± 8	<0.001
Normal Range	25-49	12 (6%)	4 (9%)	8 (5%)	
Mildly Depressed	50-59	104 (53%)	28 (62%)	76 (50%)	
Moderately Depressed	60-69	59 (30%)	11 (24%)	48 (32%)	
Severely Depressed	≥70	22 (11%)	2 (4%)	20 (13%)	0.197
**Anxiety**	**SAS**	62 ± 7	64 ± 6	62 ± 8	0.052
Normal Range	<45	1 (1%)	0 (0%)	1 (1%)	
Mild to moderate anxiety levels	45-59	59 (30%)	9 (19%)	50 (34%)	
Marked to severe anxiety levels	60-74	129 (67%)	38 (79%)	91 (62%)	
Extreme anxiety levels	≥75	5 (3%)	1 (2%)	4 (3%)	0.188
**Sleep**	**Insomnia symptoms**	122 (60%)	10 (22%)	112 (71%)	<0.001
	**OSA symptoms**	79 (39%)	11 (24%)	68 (43%)	0.018
	**Daytime Sleepiness**	71 (35%)	5 (11%)	65 (42%)	<0.001
**SDS≥60 combined with OSA or insomnia symptoms**		57 (29%)	4 (8%)	53 (35%)	<0.001

VAS: Visual Analogue Scale, QBPDS: Quebec Back Pain Disability Scale, SDS: Zung Self-Rating Depression Scale, SAS: Zung Self-Rating Anxiety Scale.
